# *MIF* mRNA Expression and Soluble Levels in Acute Coronary Syndrome

**DOI:** 10.1155/2018/9635652

**Published:** 2018-07-02

**Authors:** Emmanuel Valdés-Alvarado, Yeminia Valle, José Francisco Muñoz-Valle, Ilian Janet García-Gonzalez, Angelica Valdez-Haro, Hector Enrique Flores-Salinas, Jorge Manuel Pérez-Ibarra, Elena Sandoval-Pinto, Jorge Ramón Padilla-Gutiérrez

**Affiliations:** ^1^Instituto de Investigación en Ciencias Biomédicas, Centro Universitario de Ciencias de la Salud, Universidad de Guadalajara, Sierra Mojada 950, Col. Independencia, 44340 Guadalajara, JAL, Mexico; ^2^Centro Universitario de Ciencias de la Salud, Universidad de Guadalajara, Sierra Mojada 950, Col. Independencia, 44340 Guadalajara, JAL, Mexico; ^3^Centro Médico Nacional de Occidente (CMNO), IMSS, Independencia Oriente, 44340 Guadalajara, JAL, Mexico; ^4^Especialidad de Cardiología, Centro Médico Nacional de Occidente (CMNO), IMSS, Independencia Oriente, 44340 Guadalajara, JAL, Mexico

## Abstract

Acute coronary syndrome (ACS) describes any condition characterized by myocardial ischaemia and reduction in blood flow. The physiopathological process of ACS is the atherosclerosis where MIF operates as a major regulator of inflammation. The aim of this study was to assess the mRNA expression of MIF gene and its serum levels in the clinical manifestations of ACS and unrelated individuals age- and sex-matched with patients as the control group (CG). All samples were run using the conditions indicated in TaqMan Gene Expression Assay protocol. Determination of MIF serum levels were performed by enzyme-linked immunosorbent assay and MIF ELISA Kit. ST-segment elevation myocardial infraction (STEMI) and non-ST-segment elevation myocardial infarction (NSTEMI) showed 0.8 and 0.88, respectively, less expression of *MIF* mRNA with regard to CG. UA and STEMI presented more expression than NSTEMI 5.23 and 0.68, respectively. Otherwise, ACS patients showed significant higher MIF serum levels (*p*=0.02) compared with CG. Furthermore, the highest soluble levels of MIF were presented by STEMI (11.21 ng/dL), followed by UA (10.34 ng/dL) and finally NSTEMI patients (8.75 ng/dL); however, the differences were not significant. These novel observations further establish the process of MIF release after cardiovascular events and could support the idea of MIF as a new cardiac biomarker in ACS.

## 1. Introduction

Acute coronary syndrome (ACS) is a cardiovascular disease, which describes any condition characterized by signs and symptoms of sudden myocardial ischaemia and reduction in blood flow to the heart [1]. ACS surrounds three clinical conditions that result from an acute imbalance between myocardial oxygen supply and demand: unstable angina (UA), non-ST-segment elevation myocardial infarction (NSTEMI), and ST-segment elevation myocardial infarction (STEMI) [2, 3]. From being an illness seen predominantly in developed countries, ACS is now becoming increasingly more common in developing countries; specifically in Mexico, it is one of the main causes of death [4, 5].

The physiopathological process of ACS is the atherosclerosis, the build-up of an atherosclerotic plaque starts at lesion-prone areas in large- and medium-sized arteries where the endothelium is dysfunctional, induced by cardiovascular risk factors like chronic smoking, hypertension, and permeation of macromolecules such as lipoproteins to the intima layer [6, 7]. Dysfunctional endothelium is a key factor in atherosclerosis that favors the increase of the expression of chemotactic and adhesion molecules (such as, intercellular adhesion molecule 1, ICAM1, and vascular cell adhesion molecule 1, VCAM1, as well as E-selectin and P-selectin) and enhanced recruitment and accumulation of monocytes [6, 8, 9]. There is increasing evidence from clinical and experimental studies for a causative role of chronic inflammation in initiation and progression of atherosclerosis [10], where the release of cytokines from platelets, immune, endothelial and smooth muscle cells play a key role [10, 11]. Intracoronary ACS levels of cytokines (IL-1, IL-6, IL-8, IL-12, IL-17, TNF-*α*, tissue plasminogen activator inhibitor (tPAI)-1, and macrophage migration inhibitory factor, MIF) are increased when compared with aortic blood [9, 12, 13].

MIF is a multifunctional protein that operates as a cytokine and acts as a major regulator of inflammation and a central upstream mediator of innate and adaptive immune response. Multiple clinical studies have demonstrated the use of MIF as a biomarker for different diseases that have an inflammatory component [14, 15]. Proinflammatory actions of MIF have been reported in various inflammatory diseases such as sepsis, rheumatoid arthritis, and atherosclerosis [16, 17]. MIF was identified as a major regulator of atherogenesis by promoting the recruitment of mononuclear cells, activating inflammatory signaling pathways, and transdifferentiating macrophages into foam cells in the vessel wall as well as by enhancing collagenase expression and matrix degradation, the latter contributing to plaque destabilization [18–20]. MIF is secreted by immune cells but also from cardiac tissue; during the hypoxia and ischaemia events, the upregulation of MIF is mediated by activation of HIF-1*α* (hypoxia-inducible factor-1*α*) [21]; notably, abundant MIF protein is preformed and stored in cardiomyocytes, indicating the possibility of direct cardiac release as a source of MIF elevation following acute inflammation in cardiac events [19].

Currently, there is an important necessity in the research of new and more specific cardiac biomarkers that will be useful in the diagnostic and a better stratification in the different clinical conditions of the ACS; therefore, the aim of this study was to assess the mRNA expression of MIF gene and its serum levels in the clinical manifestations of ACS.

## 2. Materials and Methods

### 2.1. Subjects

The study group included 80 ACS unrelated patients in any of its clinical manifestations recruited from Hospital de Especialidades del Centro Médico Nacional de Occidente del Instituto Mexicano del Seguro Social (CMNO-IMSS) and classified according to the criteria of the American College of Cardiology (ACC) [22]. As a control group, 80 unrelated individuals without cardiovascular diseases were recruited from western Mexico.

The study was performed according to the ethical principles for experiments involving humans stated on the Declaration of Helsinki, and ethical approval was obtained by the Centro Universitario de Ciencias de la Salud, CUCS, UdeG (C.I. 065-2014). Informed consent was obtained from all patients for being included in the study.

### 2.2. MIF Expression Analysis

A total of 5 mL of peripheral blood was collected from all individuals in EDTA tubes, and samples from ACS patients were collected during the 24 hours after the acute event. Dextran reagent was used to the isolation of total leucocytes and trizol reagent (Invitrogen, Carlsbad, CA, USA) to the obtaining of total RNA according to the Chomiczyki and Sacchi method [23]. After the determination of purity and concentration of RNA obtained, 1 g of total RNA was reverse transcribed using reverse transcription reagents by following the manufacturer's protocol (Promega Corporation, USA). The mRNA levels of *MIF* were normalized to glyceraldehyde 3-phosphate dehydrogenase (GAPDH) (internal control) and relatively quantified by qPCR, using the 2^−∆∆Cq^ method [24]. Expression of both genes was quantified using TaqMan probes, and all samples were run in duplicate using the conditions indicated in the TaqMan Gene Expression Assay protocol in a LightCycler NanoSystem (Roche Applied Science). Changes in gene expression were expressed as a relative fold‐increase in mRNA compared with that of the control.

### 2.3. MIF Serum Levels

Serum was obtained from all individuals. The determination of MIF serum levels was performed by enzyme-linked immunosorbent assay (ELISA) and the commercial Human MIF ELISA Kit (RayBio®, USA), according to the manufacturer's instructions. MIF assay sensitivity was 6 pg/ml.

### 2.4. Statistical Analysis

All the statistical analyses were performed using SPSS software (v.18.0) and GraphPad Prism 5 software and SPSS statistical package version 21.0. Differences in characteristics between groups were analyzed using the chi-square test for categorical variables (data presented as percentages), Student's *t*-test for parametric variables (data presented as mean ± SD), and Mann–Whitney *U*-test for nonparametric variables (data presented as median ± interquartile range 25–75). *p* > 0.05 was considered statistically significant.

## 3. Results

### 3.1. Clinical and Demographic Characteristics

All clinical characteristics are shown in Table 1. The median age of CG and ACS groups was 53.5 and 63 years, respectively. The gender distribution among ACS individuals was 52% male and 48% female. The most common risk factor present in the ACS patients was high blood pressure (61.25%), followed by obesity and smoking (55% both). Also routine biochemical tests were found within reference values in patients and in the CG, except for glucose that was over the reference value in patients with ACS (124 mg/dL). About the cardiac biomarkers, we found that CK and troponine I were over the range (354 IU/mL and 0.9 ng/mL, resp.).

### 3.2. MIF Expression

**Figure 1:**
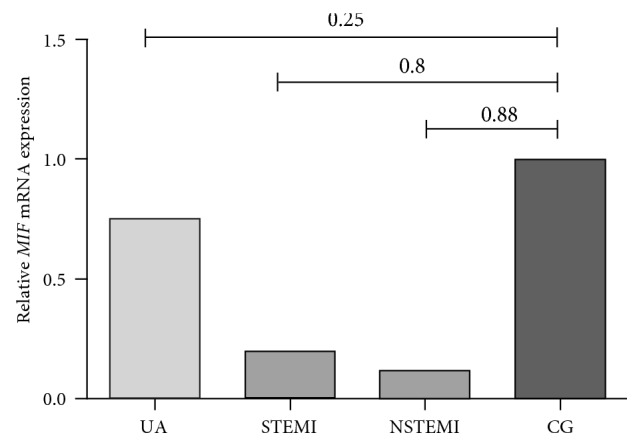
Relative *MIF* mRNA expression of total leucocytes in the clinical manifestation of ACS and CG. STEMI and NSTEMI samples showed 0.8 and 0.88, respectively, less *MIF* mRNA expression compared with CG.

**Figure 2:**
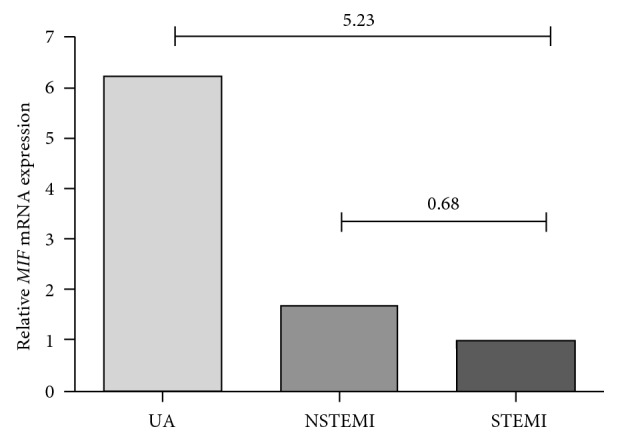
Relative *MIF* mRNA expression of total leucocytes in the clinical manifestation of ACS. UA and STEMI presented more *MIF* mRNA expression than NSTEMI (5.23 and 0.68, resp.).

Relative *MIF* mRNA expression in total leucocytes was compared between the clinical manifestations of ACS and CG Figure 1. STEMI and NSTEMI samples showed 0.8 and 0.88, respectively, less *MIF* mRNA expression compared with CG. As is already known, the severity of each ACS manifestation is different; for that reason, we also analyzed the MIF mRNA expression among UA, STEMI, and NSTEMI, Figure 2. UA and STEMI presented more expression than NSTEMI, (5.23 and 0.68, resp.).

### 3.3. MIF Soluble Levels

**Figure 3:**
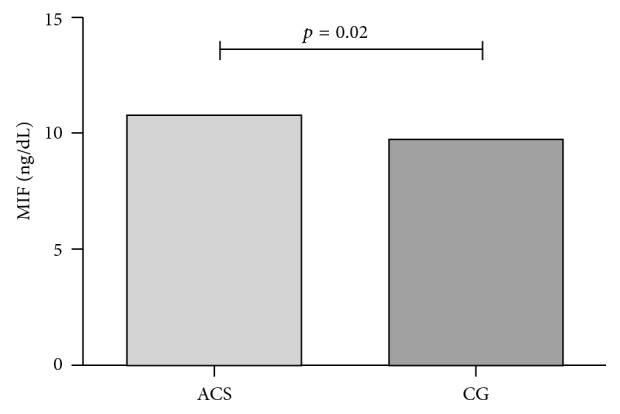
MIF soluble levels in ACS patients and CG. ACS patients showed significant higher MIF soluble levels (*p*=0.02) compared with CG (10.76 and 9.72 ng/dL, resp).

**Figure 4:**
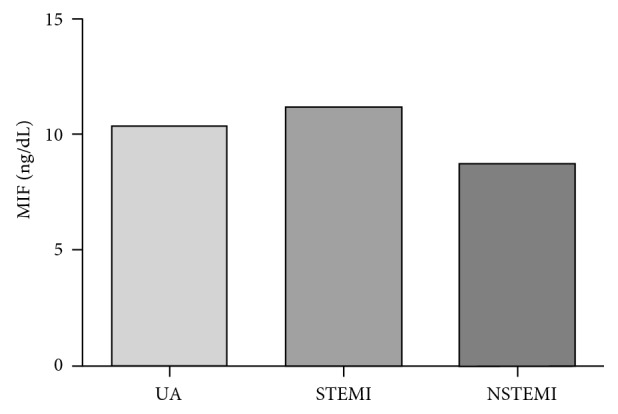
MIF soluble levels in the different clinical manifestation of disease. STEMI patients present the highest soluble levels of MIF (11.21 ng/dL), followed by UA patients (10.34 ng/dL), and finally by NSTEMI patients (8.75 ng/dL). No significant difference was shown.

MIF soluble levels were measured in ACS patients and CG (Figure 3). ACS patients showed significant higher MIF serum levels (*p*=0.02) compared with CG (10.76 and 9.72 ng/dL, resp.). Furthermore, we quantified the MIF soluble levels in the different clinical manifestation of disease (Figure 4). The highest soluble levels of MIF were presented by STEMI (11.21 ng/dL), followed by UA (10.34 ng/dL), and finally by NSTEMI patients (8.75 ng/dL); however, the differences were not significant.

## 4. Discussion

ACS is a cardiovascular disease characterized for ischaemia periods and triggered by inflammatory response [15]. The etiology of the ACS arises through a combination of environmental and genetic risk factors. Obesity, high blood pressure, dyslipidemia, diabetes mellitus type 2, obesity, and smoking are risk factors that contribute to cardiovascular risk and mortality [25–27] and are the main risk factors found in our study group. Moreover, cardiovascular biomarkers are useful for the diagnosis and management of the patients; we found in ACS patients that CK and troponine I values were over the reference value, and it is already known that their elevated levels are associated with cardiac injury and myonecrosis [28]. Regarding biochemical parameters like glucose, cholesterol, triglycerides, HDLc, and LDLc, all levels were under the reference value except glucose; it is important to highlight that ACS patients were recruited after the acute event and that they are under pharmacological treatment, specifically statins, and it is already known that the statins are a family of cholesterol-lowering drugs [26]. On the other hand, MIF is a multifunctional cytokine that acts as a major regulator of inflammation and a central upstream mediator of innate immune response [16]. Different studies have associated MIF with multiple numbers of immune and inflammatory diseases [15, 16, 29, 30]. The atheroprogressive effects of MIF can be linked with MIF's potential to trigger the expression of inflammatory mediators and mediate leukocyte recruitment and arrest directly or through the induction of adhesion molecules and chemokines in ECs and monocytes/macrophages [31]. MIF is expressed in several cell types, including monocytes, macrophages, vascular smooth muscle cells (SMCs), and cardiomyocytes [32, 33]. During events such as acute myocardial infarction, hypoxia and oxidative stress induce the release of MIF from cardiomyocytes [32]. There is evidence about the participation of MIF in atherosclerosis and ACS, but there is not much knowledge about *MIF* mRNA expression in ACS and specifically how this expression is changing between the clinical manifestations. In the present study, we unexpectedly found that *MIF* mRNA expression in STEMI and STEMI samples was decreased compared to CG. To the best of our knowledge, there are not studies about *MIF* mRNA expression in the three clinical spectrums of ACS. It is important to highlight that *MIF* expression was quantified in total leucocytes. MIF could be released during the acute event from different cellular sources, such as cardiomyocytes and leucocytes [18, 19]. Dayawansa et al. mention that upon ischaemia injury, MIF is rapidly released from the myocardium and mediates cardioprotection; however, with severe and prolonged ischaemia, elevation of MIF activates circulating peripheral blood mononuclear cells (PBMCs), promoting regional recruitment of inflammatory cells, which sustain the expression of MIF and other inflammatory molecules [18]. This information supports our findings because samples from ACS patients were collected during the 24 hours after the acute event; at this point, the release of soluble levels of MIF is mainly from cardiomyocytes, and MIF expression of PBMCs is not active yet. Furthermore, MIF expression in PBMCs is activated in autocrine way (MIF released from cardiomyocytes) and also by other inflammatory cytokines; in this regard, Dayawansa et al. published that the expression of inflammatory mediators (MIF, IL-6, MMP-9) by PBMC did not change in cells isolated from patients at hospital admission (approximately 3 h after symptom onset), but increased markedly at 3 days after myocardial infraction [18]. Regarding the *MIF* mRNA expression between UA, NSTEMI, and STEMI, we found more expression in UA compared with NSTEMI and STEMI. UA is characterized for a chronic inflammation and prolonged ischaemia periods that could led to the activation of PBMCs and the increase of *MIF* expression. Furthermore, the difference in the *MIF* expression suggests that MIF performs multiple and sometimes opposing functions depending on its cellular source, the severity of ischaemic injury, and the time after acute MI [18]. Regarding the MIF soluble levels, we found significantly higher concentration in the ACS patients than in CG (*p*=0.02). The findings of our research are in accordance with previous studies demonstrating the effect of MIF on the ACS and atherosclerosis [13, 18, 19, 31, 32] and with the other research published by Van der Vorst et al. that showed that serum MIF levels in cardiovascular disease (CDV) patients were associated with inflammatory markers like CRP and IL-6 and also as a high independent risk factor for future coronary events in patients with CVD [34]. Furthermore, in the setting of ischaemia or hypoxia, upregulation of MIF is mainly mediated by activation of HIF-1*α* (hypoxia-inducible factor-1*α*) and not by other proinflammatory cytokines as TNF-*α* [21]. As MIF has been associated with infarct size [19, 32], we compared the cytokine serum levels in UA, STEMI, and NSTEMI groups without significant differences. We observed only a tendency in STEMI and NSTEMI groups that express more MIF serum levels than UA, and these results are in concordance with the previous studies published by Chan et al. and Yüksel et al. that propose a correlation between the infarct size and MIF serum levels [32, 35]. A critical step for the patients' health outcome needs an early and accurate diagnosis; now a days, symptoms, vital signs, electrocardiograms, and various lab assays may be used to help triage patients toward discharge, continued monitoring, medical therapy, and invasive interventions [28]. Also the lab assays focus on the search of cardiac biomarkers such as troponins, creatine kinase-MB, myoglobin, and brain natriuretic peptide. Nevertheless, studies about increase in MIF serum levels post-MI and its relation with infraction size suggest a role of MIF as a possible cardiac biomarker in ACS.

## 5. Conclusion

These novel observations further establish the process of MIF release after cardiovascular events and could support the idea of MIF as a new cardiac biomarker in patients with coronary events. Furthermore, the function of MIF after the acute event depends on the cell source of delivery. Nevertheless, future studies are necessary to elucidate the complete mechanism of MIF and its implication in the development and progression and its role after the ACS.

## Figures and Tables

**Table 1:** Demographic and clinical data of the CG and patients with ACS.

	CG median (IQR 25–75) *n*=80	ACS median (IQR 25–75) *n*=80	Reference value	
Age (years)	53.5 ± 13	63 ± 11	—	
Male/female	39/41	42/38	—	
Glucose (mg/dL)	89 (76–102)	124 (102–226)	75–100	
Cholesterol (mg/dL)	148 (123–168)	115 (97–154)	<200	
Triglycerides (mg/dL)	86 (37–200)	88 (73–123)	<200	
HDLc (mg/dL)	24 (13–33)	16 (13–23)	<60	
LDLc (mg/dL)	55 (45–78)	39 (32–61)	<129	
CK (IU/mL)	—	354 (109–690)	24–195	
CK-MB (IU-mL)	—	36 (20–91)	<130	
Troponine I (ng/mL)	—	0.9 (0.1–3.14)	0.1–0.4	
hs-CPR (mg/L)	18 (3–36)	3 (1.6–3.9)	6.6–8.5	

*Risk factor*	*n (%)*	*n (%)*	*ACS diagnosis n (%)*	
Obesity	20 (25)	44 (55)	UA (32.5)	26
Diabetes mellitus type 2	4 (5)	40 (50)	STEMI (33.75)	27
Dyslipidemia	2 (1.6)	35 (43.75)		
High blood pressure	16 (20)	49 (61.25)	NSTEMI (33.75)	27
Smoking	4 (5)	44 (55)		

ACS: acute coronary syndrome; CG: control group; CK: creatine kinase; CK-MB: creatine kinase muscle and brain; HDLc: high density lipoprotein; IQR: interquartile range; LDLc: low density lipoprotein; NSTEMI: non-ST-segment elevation myocardial infarction; UA: unstable angina; and STEMI: ST-segment elevation myocardial infarction (STEMI). The upper limit of normal CK is defined by individual hospital laboratory standards.

## Data Availability

The datasets used and/or analyzed during the current study are available from the corresponding author on reasonable request.
